# Supplementary material for: TrkB agonist antibody ameliorates fertility deficits in aged and cyclophosphamide-induced premature ovarian failure model mice
Source: Nat Commun. 2022 Feb 17;13:914. doi: 10.1038/s41467-022-28611-2 (PMC8854395; doi:10.1038/s41467-022-28611-2)
Supplement: Supplementary file 1 — Supplementary information [file 41467_2022_28611_MOESM1_ESM.pdf]

Supplementary information for:

TrkB agonist antibody ameliorates fertility deficits in aged and cyclophosphamide-induced premature ovarian failure model mice

Qin et al.

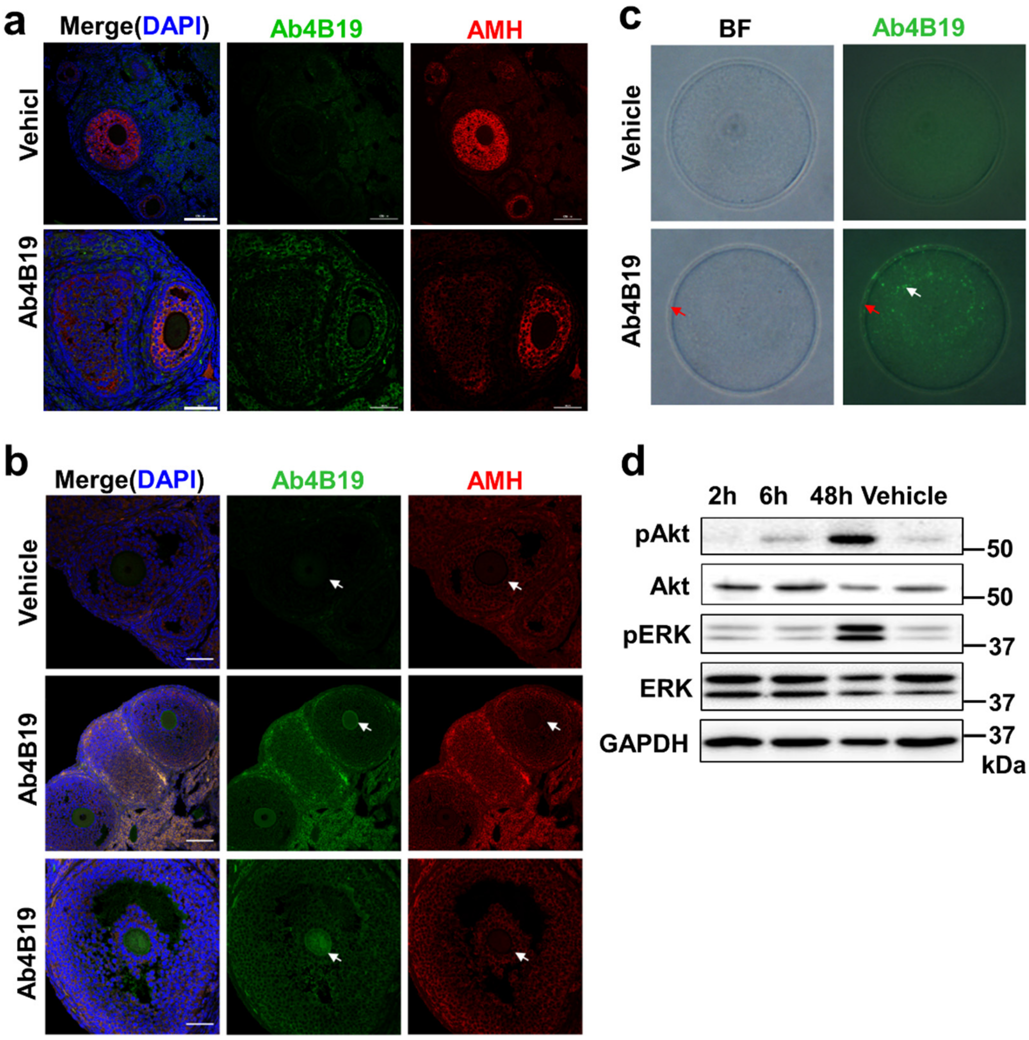

Supplementary Fig. 1 Detection of Ab4B19 in follicles after its iv administration. The rabbit antibody Ab4B19 was detected with anti-rabbit IgG (FITC, green); AMH was probed with mouse anti-AMH and detected with anti-mouse IgG (TRITC, red). Cell nucleus were labeled with DAPI (blue). **a** Immunostaining images showing Ab4B19 penetration into follicles 24 hours (24h) after tail-vein injection. Scale bar, 100 µm. **b** Representative images depicting the enrichment of Ab4B19 around oocytes 48 hours (48h) after its administration. White arrowheads point to the localization of Ab4B19 on an oocyte in ovarian follicle. Scale bar, 200 µm. Experiments were repeated 3 times independently with similar results (4 sections/mice each time). **c** Representative images of oocytes treated with Ab4B19 cultured for 12 hours. Ab4B19 was detected with anti-rabbit IgG (FITC, green). BF: bright field. White arrow indicates the presence of Ab4B19 in oocyte cytoplasm. Red arrow indicates zona pellucida. Experiments were repeated 3 times independently with similar results. **d** Time course of TrkB activation in ovary after Ab4B19 administration (1 mg/kg; iv) into 8-week-old mice. Ovary tissues were collected and lysed at different time points after tail vein injection of Ab4B19, and Western blots were performed to detect pAkt and pERK. Experiments were repeated at least two times with similar results. Source data are provided as a Source Data file.

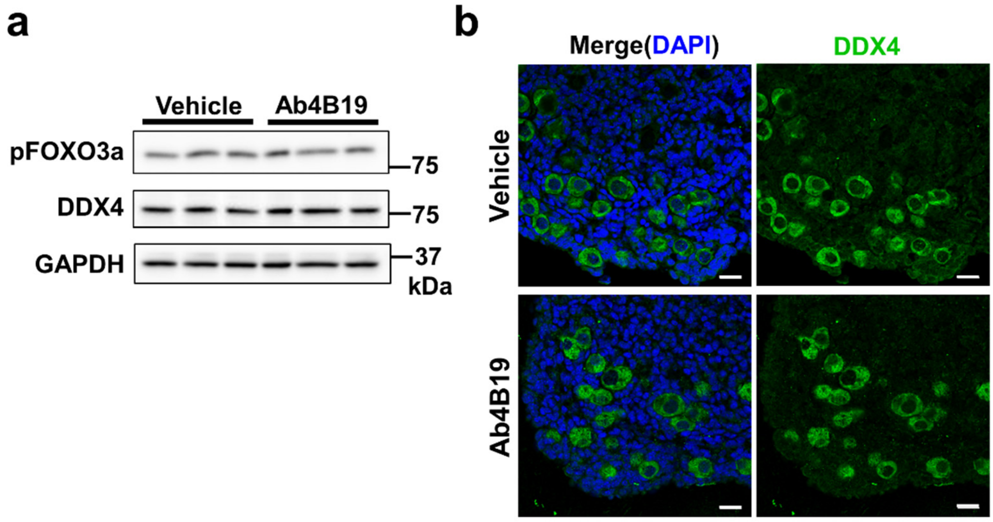

Supplementary Fig. 2 Effect of Ab4B19 on primordial follicles. a Representative Western blot of pFOXO3a and DDX4 expression in cultured P3 ovaries after administration with Ab4B19 for 48 hours. **b** Immunofluorescence staining showing the expression DDX4 for experiment of (**a**). (Green: DDX4, Blue: DAPI). Scale bars, 20 μm. The experiment was repeated three times. Source data are provided as a Source Data file.

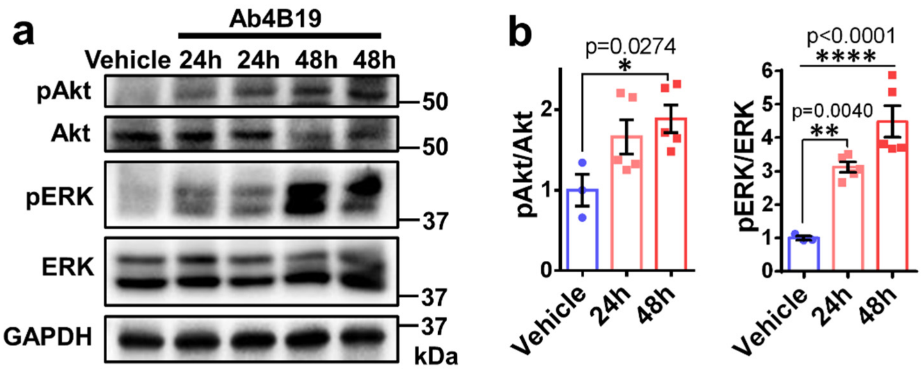

Supplementary Fig. 3 Activation of TrkB signaling pathways in NA-POF model. a Phosphorylated and total Akt, Erk were measured by Western blotting with GAPDH used as a loading control, at 24 hours and 48 hours after Ab4B19 treatment. **b** The ratios of phosphorylated/total proteins were calculated and plotted. A total of 13 mice were used, with tissues from 5 mice for each time point and 3 mice for Vehicle. Data are presented as mean ± SEM. Statistical analyses were carried out using one-way ANOVA followed by the Dunnett's multiple comparisons test. (*P < 0.05; **P < 0.01; ****P < 0.0001). Source data are provided as a Source Data file.

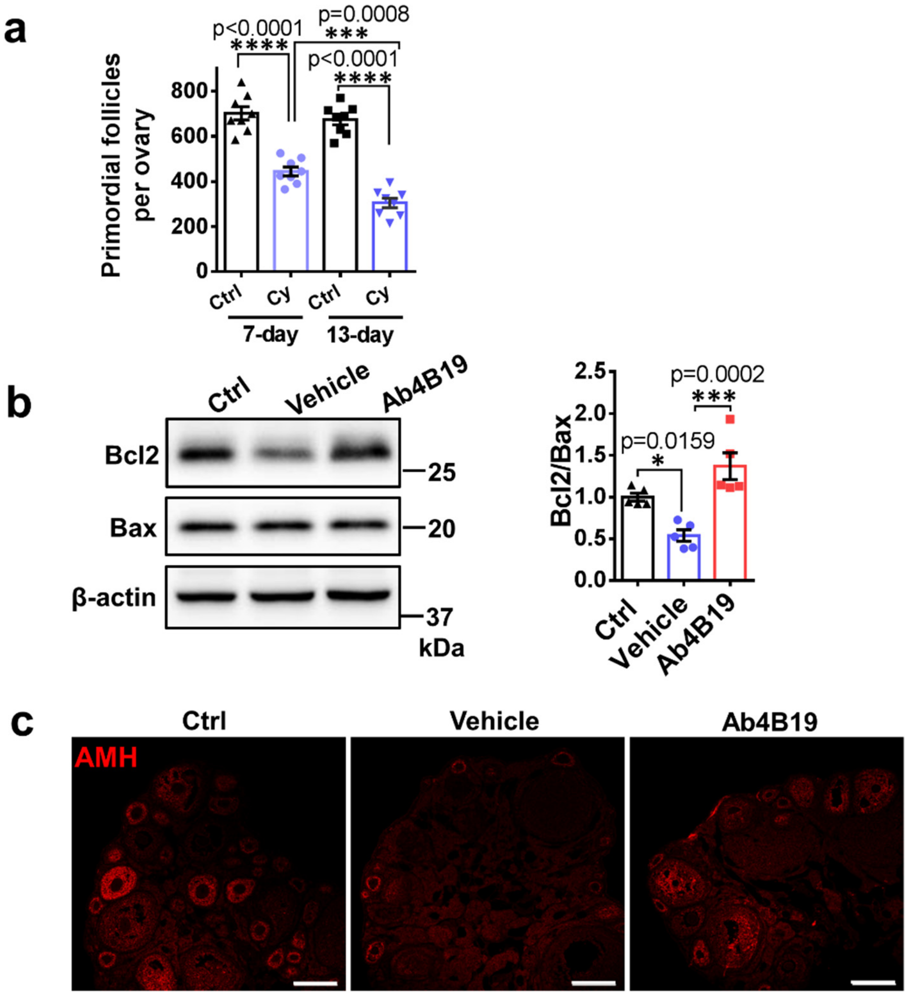

Supplementary Fig. 4 Reversal of gonadotoxicity and AMH expression by Ab4B19 in Cy-POF model. a Quantification of primordial follicles in normal mice (Ctrl) and Cy-POF mice. Mice were treated with vehicle or 75mg/kg Cy through intraperitoneal injection. Ovaries were collected for immunostaining 7 or 13 days after Cy treatment. N = 8 ovaries per each condition. **b** Inhibition of apoptosis by Ab4B19 in Cy-POF mice. Cy-POF mice were treated with Ab4B19 as shown in Fig. 6a. The lysates of ovarian tissues were collected and processed for Western blotting to detect the expression of Bcl2 and Bax. The blots were quantified and relative intensities of Bcl2/Bax are shown in the left panel. N = 5 mice per group. Data are presented as mean ± SEM. Statistical analyses were carried out using one-way ANOVA, followed by Dunnett's multiple comparisons test (**a**, **b**). (*$P < 0.05$; **$P < 0.01$; ***$P < 0.001$; ****$P < 0.0001$). Source data are provided as a Source Data file. **c** Immunofluorescent images showing the expression of AMH in ovaries in normal (Ctrl), and Cy-POF mice treated with vehicle or Ab4B19 for 6 days. Scale bars, 200 µm. Experiments were repeated at least 3 times independently with similar results (4 sections/mice each time).

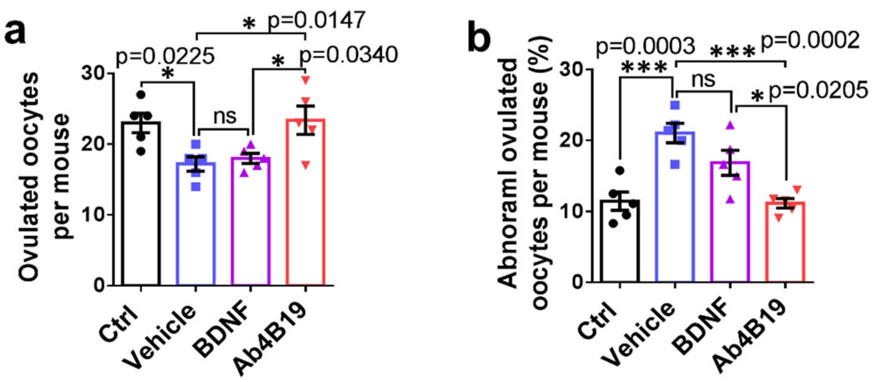

Supplementary Fig. 5 a-b Quantification of ovulated oocytes and abnormal oocytes in Cy-POF mice treated with BDNF or Ab4B19. With the non-Cy treated mice as Ctrl, Cy-POF mice were treated with normal IgG (Vehicle), BDNF (0.17 mg/kg/2d) and Ab4B19 (1 mg/kg/3d) mice for 6 days. The number of ovulated oocytes (left) and the ratio of abnormal oocytes/total ovulated oocytes (right) were quantified (N = 5 mice per group). Note: 0.17 mg/kg BDNF (MW: ~25kD) is same as 1 mg/kg Ab4B19 (MW: ~150KD) in molar unit. Data are presented as mean ± SEM. Statistical analyses were carried out using one-way ANOVA followed by the Dunnett's multiple comparisons test. (*P < 0.05; **P < 0.01; ***P < 0.001). Source data are provided as a Source Data file.

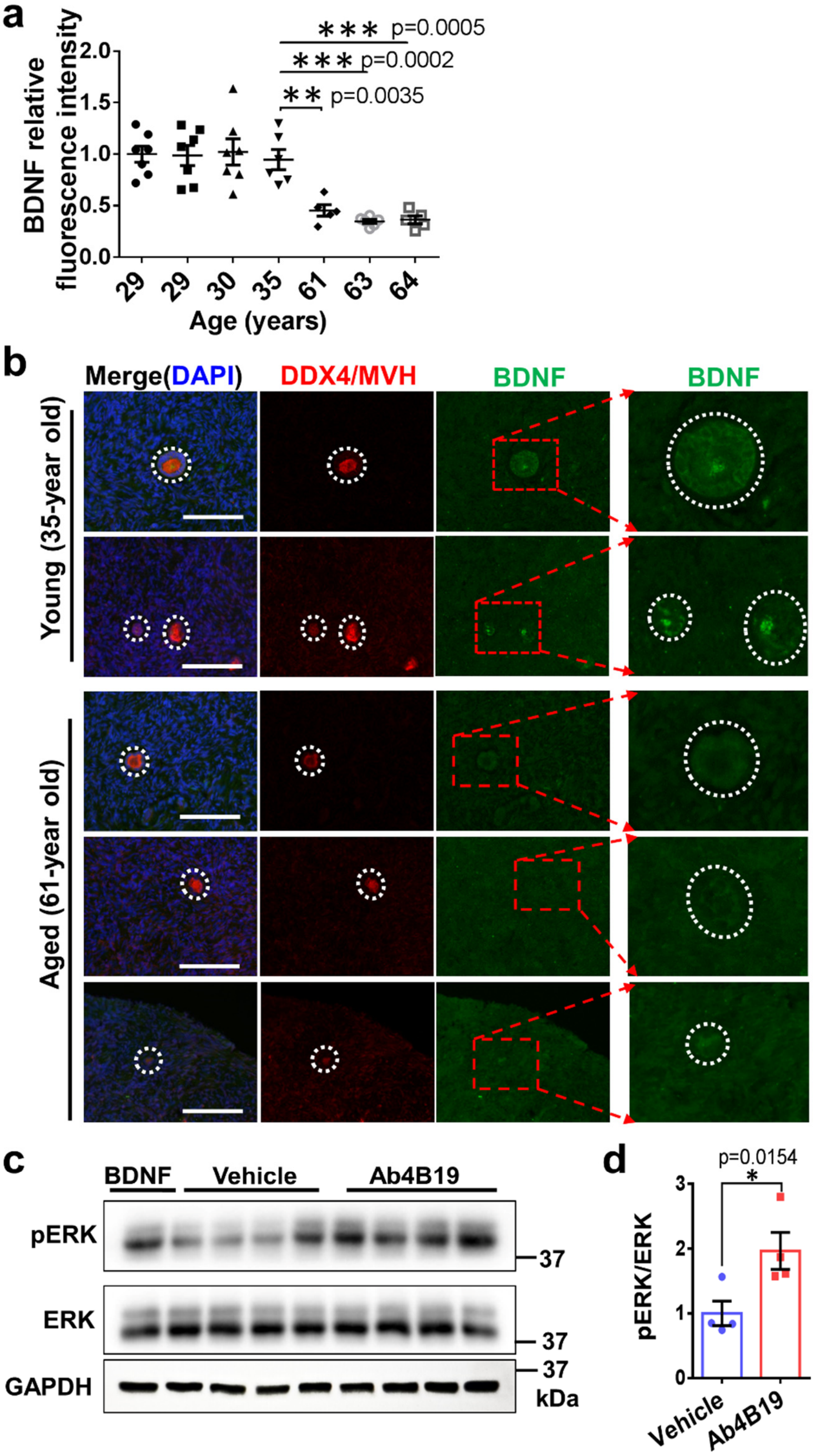

Supplementary Fig. 6 a BDNF expression in human ovary at different ages. n = 7 ovarian follicles in 29-year and 30-year old groups, 6 in 35-year and 63-year old groups, and 5 in 61-year and 64-year old groups. BDNF-immunoreactivity (relative fluorescence intensity) of ovarian paraffin sections derived from 29-73 years old women was detected by a rabbit anti-BDNF antibody followed by anti-rabbit IgG (FITC, green) and co-labelled with a mouse anti-DDX4/MVH antibody followed by anti-mouse IgG (TRITC, red). At least five sections of each age were stained and examined. Small follicles (diameter<100 μm) were captured and relative fluorescence intensities

were quantified using Image J. **b** Representative BDNF and DDX4/AMH staining of small follicles from a 35-year-old young and a 61-year-old aged females in (**a**). The outlines of follicles were marked using dotted white circles. Scale bars, 100 μm. **c** Activation of ERK signaling pathways in human ovary. Ovarian tissues from pre-menopausal women (within 1 hour after the surgery) were treated with 1 nM BDNF, 3 nM normal IgG or 3 nM Ab4B19 for 35 minutes. Phosphorylated and total Erk were measured by Western blotting with GAPDH used as a loading control. **d** The ratios of phosphorylated/total proteins were calculated and plotted after BDNF or Ab4B19 treatment. N = 4 tissues per group. Data are all presented as mean ± SEM. Statistical analyses were carried out using one-way ANOVA followed by the Dunnett's multiple comparisons test (**a**), or two-tailed student's t-test (**d**), respectively (*$P < 0.05$; **$P < 0.01$; ***$P < 0.001$). Source data are provided as a Source Data file.

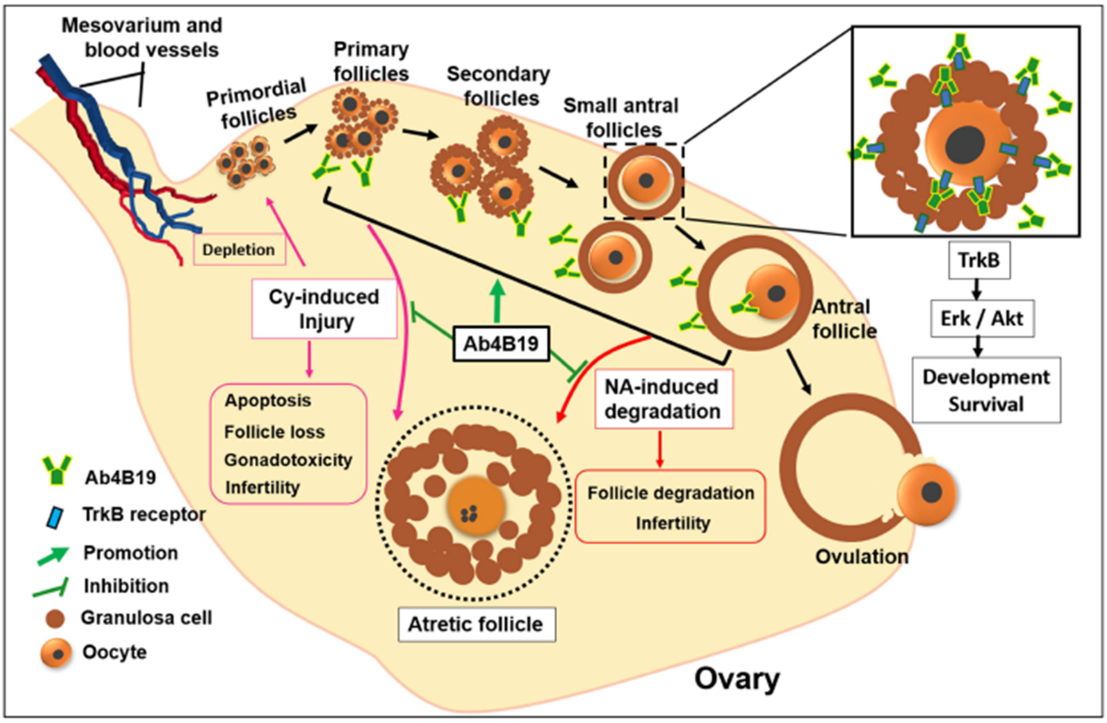

Supplementary Fig. 7 Schematic diagram showing the proposed therapeutic effects of Ab4B19 on folliculogenesis in NA- and Cy-induced POF models. NA- and Cy-induced POF models were used to assess the therapeutic potential of the TrkB antibody Ab4B19. Through tail vein injection, Ab4B19 flowed into the ovary and penetrated into the ovarian follicles. There, it maintained/promoted follicle development via activation of TrkB signaling. In NA-POF model, treatment with Ab4B19 rescued follicle degradation and infertility. Similarly, In Cy-POF model, Ab4B19 reversed ovarian follicle loss, repaired gonadotoxicity, and restored infertility.

Supplementary Table 1. Summary of fertility parameters for first-generation mice from NA-POF mice treated with or without Ab4B19.

Items	NA-POF	NA-POF+Ab4B19	*P*
Fertility index # (fraction of females that delivered offspring/total)	4/4(100%)	6/6(100%)	—
Estrous cycles*	normal	normal	—
Number of pups*	9.250 ± 0.4787	9.667 ± 0.5578	0.2933
Mean body weight of pups (g) *	1.276 ± 0.01713	1.292 ± 0.01911	0.2809

In this and all other tables, data represent the mean ± SEM. #Statistical analysis was performed using Chi-square test. *Statistical analyses were performed by two-tailed Student's *t*-test. Source data are provided as a Source Data file.

Supplementary Table 2. Health status of first-generation mice derived from the NA-POF mice treated with or without Ab4B19.

Items	Aging mice	Aging mice+Ab4B19	*P*
TC, mM, (female)	1.660 ± 0.06633	1.707 ± 0.1474	0.3906
TC, mM, (male)	2.318 ± 0.03774	2.216 ± 0.1050	0.2012
LDL, mM, (female)	0.215 ± 0.02363	0.175 ± 0.0117	0.0858
LDL, mM, (male)	0.304 ± 0.00927	0.306 ± 0.0320	0.4773
HDL, mM, (female)	1.577 ± 0.07107	1.570 ± 0.1346	0.4831
HDL, mM, (male)	1.836 ± 0.07215	1.800 ± 0.05621	0.3524
TG, mM, (female)	0.200 ± 0.02683	0.290 ± 0.06356	0.1175
TG, mM, (male)	0.584 ± 0.07652	0.746 ± 0.1677	0.2078
GLU, mM, (female)	4.505 ± 0.5485	4.207 ± 0.5082	0.3492
GLU, mM, (male)	15.42 ± 0.4956	15.22 ± 0.4310	0.3854
FSH, ng/ml, (female)	8.476 ± 1.211	7.907 ± 1.174	0.3714
LH, ng/ml, (female)	1.830 ± 0.2537	1.753 ± 0.1168	0.3949
Ovary morphology^	normal	normal	—
Testis morphology^	normal	normal	—
Liver morphology^	normal	normal	—

TC, total cholesterol; LDL-C, low density lipoprotein cholesterol; HDL-C, high density lipoprotein cholesterol; TG, triglyceride; GLU, glucose; FSH, follicle-stimulating hormone; LH, luteinizing hormone. ^Morphology analysis was performed by histological assessment of H&E staining. Statistical analyses were performed by two-tailed Student's *t*-test. Source data are provided as a Source Data file.

Supplementary Table 3. Summary of fertility parameters for the second-generation mice derived from the NA-POF mice treated with or without Ab4B19.

Items	Aging mice	Aging mice+Ab4B19	*P*
Fertility index [#]<br>(fraction of females<br>that delivered offspring/total)	6/6(100%)	6/6(100%)	—
Estrous cycles[*]	normal	normal	—
Number of pups[*]	9.500 ± 0.5627	9.333 ± 0.4216	0.4089
Mean body weight of pups (g) [*]	1.262 ± 0.01778	1.296 ± 0.01787	0.1034

Statistical analyses were performed by two-tailed Student's *t*-test. Source data are provided as a Source Data file.

Supplementary Table 4. Health status of the second-generation mice derived from the NA-POF mice treated with or without Ab4B19.

Items	Aging mice	Aging mice+Ab4B19	*P*
TC, mM, (female)	2.233 ± 0.3867	2.235 ± 0.2653	0.4986
LDL, mM, (female)	0.2050 ± 0.0211	0.1783 ± 0.008724	0.1414
HDL, mM, (female)	1.948 ± 0.2937	1.947 ± 0.1981	0.4982
TG, mM, (female)	0.4083 ± 0.083	0.4017 ± 0.09728	0.4797
GLU, mM, (female)	5.347 ± 1.343	5.470 ± 1.036	0.4718
Ovary morphology^	normal	normal	—
Testis morphology^	normal	normal	—
Liver morphology^	normal	normal	—

TC, total cholesterol; LDL-C, low density lipoprotein cholesterol; HDL-C, high density lipoprotein cholesterol; TG, triglyceride; GLU, glucose; FSH, follicle-stimulating hormone; LH, luteinizing hormone. ^Morphology analysis was performed by histological assessment of H&E staining. Statistical analyses were performed by two-tailed Student's *t*-test. Source data are provided as a Source Data file.

Supplementary Table 5. Health status of NA-POF mice treated with or without Ab4B19.

Items	Aging mice	Aging mice+Ab4B19	*P*
TC, mM, (female)	2.267 ± 0.1775	2.153 ± 0.1443	0.6607
LDL, mM, (female)	0.1983 ± 0.01138	0.1833 ± 0.008819	0.5898
HDL, mM, (female)	1.098 ± 0.05935	1.170 ± 0.07979	0.5317
TG, mM, (female)	0.4883 ± 0.05029	0.4617 ± 0.04324	0.7481
GLU, mM, (female)	6.970 ± 0.6099	7.102 ± 0.5925	0.9509
Liver morphology^	normal	normal	—

TC, total cholesterol; LDL-C, low density lipoprotein cholesterol; HDL-C, high density lipoprotein cholesterol; TG, triglyceride; GLU, glucose; FSH, follicle-stimulating hormone; LH, luteinizing hormone. ^Morphology analysis was performed by histological assessment of H&E staining. Statistical analyses were performed by two-tailed Student's *t*-test. Source data are provided as a Source Data file.

Supplementary Table 6. Analysis of fertility parameters for the first-round mating from Cy-POF mice treated with or without Ab4B19.

Items	Ctrl	Vehicle	Ab4B19
Mating index	6/6(100%)	6/6(100%)	6/6(100%)
Fertility index[#]<br>(fraction of females that delivered offspring/total)	6/6(100%)	6/6(100%)	6/6(100%)
Mean body weight of pups (g) ※	1.317± 0.01334	1.283 ± 0.02999	1.305 ± 0.03360

※Statistical analyses were performed by one-way ANOVA followed by Tukey's multiple-comparison tests. Source data are provided as a Source Data file.
