# Supplementary material for: TrkB agonist antibody ameliorates fertility deficits in aged and cyclophosphamide-induced premature ovarian failure model mice
Source: Nat Commun. 2022 Feb 17;13:914. doi: 10.1038/s41467-022-28611-2 (PMC8854395; doi:10.1038/s41467-022-28611-2)
Supplement: Supplementary file 3 — Reporting Summary [file 41467_2022_28611_MOESM3_ESM.pdf]

nature research

Corresponding author(s): Bai Lu, Wei Guo

Last updated by author(s): Dec 25, 2021

Reporting Summary

Nature Research wishes to improve the reproducibility of the work that we publish. This form provides structure for consistency and transparency in reporting. For further information on Nature Research policies, see our Editorial Policies and the Editorial Policy Checklist.

Statistics

For all statistical analyses, confirm that the following items are present in the figure legend, table legend, main text, or Methods section.

n/a	Confirmed	
☐	☒	The exact sample size (n) for each experimental group/condition, given as a discrete number and unit of measurement
☐	☒	A statement on whether measurements were taken from distinct samples or whether the same sample was measured repeatedly
☐	☒	The statistical test(s) used AND whether they are one- or two-sided<br>*Only common tests should be described solely by name; describe more complex techniques in the Methods section.*
☒	☐	A description of all covariates tested
☐	☒	A description of any assumptions or corrections, such as tests of normality and adjustment for multiple comparisons
☐	☒	A full description of the statistical parameters including central tendency (e.g. means) or other basic estimates (e.g. regression coefficient) AND variation (e.g. standard deviation) or associated estimates of uncertainty (e.g. confidence intervals)
☐	☒	For null hypothesis testing, the test statistic (e.g. F, t, r) with confidence intervals, effect sizes, degrees of freedom and P value noted<br>*Give P values as exact values whenever suitable.*
☒	☐	For Bayesian analysis, information on the choice of priors and Markov chain Monte Carlo settings
☒	☐	For hierarchical and complex designs, identification of the appropriate level for tests and full reporting of outcomes
☒	☐	Estimates of effect sizes (e.g. Cohen's d, Pearson's r), indicating how they were calculated

Our web collection on statistics for biologists contains articles on many of the points above.

Software and code

Policy information about availability of computer code

Data collection	The commercial softwares: Tanon MP (v1.02) for Western blot, Zeiss Zen (v2.1) for imaging, and GEN5 CHS (v2.05) of Biotek for ELISA.
Data analysis	Tanon Gis (v4.2), Image J (v1.52i), GraphPad Prism (v7.0), Excel 2013 was used for data analysis.

For manuscripts utilizing custom algorithms or software that are central to the research but not yet described in published literature, software must be made available to editors and reviewers. We strongly encourage code deposition in a community repository (e.g. GitHub). See the Nature Research guidelines for submitting code & software for further information.

Data

Policy information about availability of data

All manuscripts must include a data availability statement. This statement should provide the following information, where applicable:

- Accession codes, unique identifiers, or web links for publicly available datasets
- A list of figures that have associated raw data
- A description of any restrictions on data availability

All relevant data and code are available within the article and its supplementary information/Source data or freely available from the corresponding authors upon reasonable request. The accession number for the RNA-Seq data is in the NCBI Gene Expression Omnibus (GEO) under the accession number: GSE107746 (https://www.ncbi.nlm.nih.gov/geo/query/acc.cgi?acc=GSE107746). RNA-Seq data for bdnf and trkb expression (Figure 7c-e) were also available in Source data. Source data are provided with this paper.

Field-specific reporting

Please select the one below that is the best fit for your research. If you are not sure, read the appropriate sections before making your selection.

- [x] Life sciences
- [ ] Behavioural & social sciences
- [ ] Ecological, evolutionary & environmental sciences

For a reference copy of the document with all sections, see nature.com/documents/nr-reporting-summary-flat.pdf

Life sciences study design

All studies must disclose on these points even when the disclosure is negative.

Sample size	Sample sizes were chosen based on prior knowledge from previous experiments or preliminary data demonstrating statistically significant differences for each specific assay.<br>Relevant references:<br>1. Wu, Y.-y. et al. Protective roles and mechanisms of polysaccharides from Dendrobium officinal on natural aging-induced premature ovarian failure. Biomed. Pharmacother. 101, 953-960 (2018).<br>2. Ding, C., Zou, Q., Wang, F., Wu, H., Wang, W., Li, H., and Huang, B. (2018). HGF and BFGF Secretion by Human Adipose-Derived Stem Cells Improves Ovarian Function During Natural Aging via Activation of the SIRT1/FOXO1 Signaling Pathway. Cellular physiology and biochemistry : international journal of experimental cellular physiology, biochemistry, and pharmacology 45, 1316-1332.<br>3.Tamura, H., Kawamoto, M., Sato, S., Tamura, I., Maekawa, R., Taketani, T., Aasada, H., Takaki, E., Nakai, A., Reiter, R.J., et al. (2017). Long-term melatonin treatment delays ovarian aging. Journal of pineal research 62.<br>4. Pascuali, N., Scotti, L., Di Pietro, M., Oubiña, G., Bas, D., May, M., Gómez Muñoz, A., Cuasnicú, P.S., Cohen, D.J., Tesone, M., et al. (2018). Ceramide-1-phosphate has protective properties against cyclophosphamide-induced ovarian damage in a mice model of premature ovarian failure. Human reproduction (Oxford, England) 33, 844-859.<br>5. Kamarzaman, S., Shaban, M. & Rahman, S. A. The prophylactic effect of Nigella sativa against cyclophosphamide in the ovarian follicles of matured adult mice: a preliminary study. JAPS, Journal of Animal and Plant Sciences 24, 81-88 (2014).<br>6. Roness, H. et al. Pharmacological administration of recombinant human AMH rescues ovarian reserve and preserves fertility in a mouse model of chemotherapy, without interfering with anti-tumoural effects. J. Assist. Reprod. Genet. 36, 1793-1803 (2019).
Data exclusions	No data were excluded.
Replication	Both in vitro and in vivo experiments were performed with at least 3 biological replicates.The numbers of replicates performed for each experiment are indicated in the Figure Legends. All results were reproducible.
Randomization	For the animal studies, mice with the same genetic background were randomly allocated into different experimental groups. For cell culture experiments, cells were counted and equal number of cells were seeded (allocated) randomly in culture dishes for each experimental group set up for drug treatment.
Blinding	The investigators are blind to group allocation.

Reporting for specific materials, systems and methods

We require information from authors about some types of materials, experimental systems and methods used in many studies. Here, indicate whether each material, system or method listed is relevant to your study. If you are not sure if a list item applies to your research, read the appropriate section before selecting a response.

Materials & experimental systems

n/a	Involved in the study	
	x	Antibodies
	x	Eukaryotic cell lines
x		Palaeontology and archaeology
	x	Animals and other organisms
	x	Human research participants
x		Clinical data
x		Dual use research of concern

Methods

n/a	Involved in the study	
x		ChIP-seq
x		Flow cytometry
x		MRI-based neuroimaging

Antibodies

Antibodies used	GAPDH (Mouse monoclonal antibody, Bioeasy (Beijing) Technology, #BE0023, 1:3000),<br>β-actin (Mouse monoclonal antibody, Beyotime, #AA128, 1:1000),<br>Akt (Mouse monoclonal antibody, Santa Cruz Biotechnology, #sc-81434, 1:1000),<br>phospho-Akt (Ser473) (Rabbit polyclonal antibody, Cell Signaling Technology, #9271, 1:2000),<br>TrkB (80E3) (Rabbit monoclonal antibody, Cell Signaling Technology, #4603, 1:1000),

phospho-TrkB (Rabbit monoclonal antibody, Cell Signaling Technology, #4621, 1:1000),
ERK (Rabbit monoclonal antibody, Cell Signaling Technology, #4695, 1:1000),
phospho-ERK (Rabbit monoclonal antibody, Cell Signaling Technology, #4370, 1:2000),
Cleaved Caspase-3 (Rabbit monoclonal antibody, Cell Signaling Technology, #9664, 1:1000),
DDX4 / MVH antibody (Rabbit polyclonal antibody, abcam, #ab13840, 1:1000),
BAX (Rabbit monoclonal antibody, abcam, #ab32503, 1:2000),
CREB (Rabbit monoclonal antibody, Cell Signaling Technology, #9197, 1:1000),
phospho-CREB (Rabbit monoclonal antibody, Cell Signaling Technology, #9198, 1:1000),
phospho-FOXO3a (Thr32) (Rabbit antibody, Cell Signaling Technology, #9464, 1:1000),
AMH (Rabbit polyclonal to AMH-C-terminal, abcam, #ab229212, 1:1000),
AMH (Mouse monoclonal antibody, Santa Cruz Biotechnology, #sc-6886, 1:400),
FSHR (Rabbit polyclonal antibody, Bioworld, # BS6610, 1:1000),
Bcl-2 (Mouse monoclonal antibody, Santa Cruz Biotechnology, #sc-7382, 1:800),
HRP conjugated rabbit IgG (Cell Signaling Technology, #7074, 1:4000),
mouse IgG (Abcam, #ab131368, 1:1000; Cell Signaling Technology, #7076, 1:4000),
Alexa Fluor 546 conjugated goat anti-mouse secondary antibody (Thermo Fisher Scientific, # A-11030, 2 μg/ml),
Alexa Fluor 488 conjugated goat anti-rabbit secondary antibody (Thermo Fisher Scientific, # A-11008, 2 μg/ml),
BDNF (Rabbit monoclonal antibody, abcam, #ab108319, 1:100),

Validation

GAPDH (Mouse monoclonal antibody, Bioeasy (Beijing) Technology, #BE0023, 1:3000), http://www.bioeasytech.com/home/product/article/id/7/sear/GAPDH.html
β-actin (Mouse monoclonal antibody, Beyotime, #AA128, 1:1000), https://www.beyotime.com/product/AA128.htm
Akt (Mouse monoclonal antibody, Santa Cruz Biotechnology, #sc-81434, 1:1000), https://www.scbt.com/zh/p/akt1-2-3-antibody-5c10 validated against serum starved cells
phospho-Akt (Ser473) (Rabbit polyclonal antibody, Cell Signaling Technology, #9271, 1:2000), https://www.cellsignal.com/products/primary-antibodies/phospho-akt-ser473 antibody/9271, validated against starved cells
TrkB (80E3) (Rabbit monoclonal antibody, Cell Signaling Technology, #4603, 1:1000),
https://www.cellsignal.com/products/primary-antibodies/trkb-80e3-rabbit-mab/4603, validated against neonatal mouse brain
phospho-TrkB (Rabbit monoclonal antibody, Cell Signaling Technology, #4621, 1:1000), https://www.cellsignal.cn/products/primary-antibodies/phospho-trka-tyr674-675-trkb-tyr706-707-c50f3-rabbit-mab/4621?site-search-type=Products&N=4294956287&Ntt=ptrkb&fromPage=plp, validated against starved cells
ERK (Rabbit monoclonal antibody, Cell Signaling Technology, #4695, 1:1000),
https://www.cellsignal.com/products/primary-antibodies/p44-42-mapk-erk1-2-137f5-rabbit-mab/4695
phospho-ERK (Rabbit monoclonal antibody, Cell Signaling Technology, #4370, 1:2000),
https://www.cellsignal.com/products/primary-antibodies/phospho-p44-42-mapk-erk1-2-thr202-tyr204-d13-14-4e-xp-rabbit-mab/4370
Cleaved Caspase-3 (Rabbit monoclonal antibody, Cell Signaling Technology, #9664, 1:1000),
https://www.cellsignal.com/products/primary-antibodies/cleaved-caspase-3-asp175-5a1e-rabbit-mab/9664?site-search-type=Products&N=4294956287&Ntt=caspase3&fromPage=plp
DDX4 / MVH antibody (Rabbit polyclonal antibody, abcam, #ab13840, 1:1000),
https://www.abcam.com/ddx4--mvh-antibody-ab13840.html, validated against knockout
BAX (Rabbit monoclonal antibody, abcam, #ab32503, 1:2000),
https://www.abcam.com/bax-antibody-e63-ab32503.html, validated against knockout
CREB (Rabbit monoclonal antibody, Cell Signaling Technology, #9197, 1:1000),
https://www.cellsignal.com/products/primary-antibodies/creb-48h2-rabbit-mab/9197?site-search-type=Products&N=4294956287&Ntt=creb&fromPage=plp, validated using SimpleChIP® Enzymatic Chromatin IP Kits.
phospho-CREB (Rabbit monoclonal antibody, Cell Signaling Technology, #9198, 1:1000),
https://www.cellsignal.com/products/primary-antibodies/phospho-creb-ser133-87g3-rabbit-mab/9198?site-search-type=Products&N=4294956287&Ntt=phospho-creb&fromPage=plp, validated using SimpleChIP® Enzymatic Chromatin IP Kits.
phospho-FOXO3a (Thr32) (Rabbit antibody, Cell Signaling Technology, #9464, 1:1000), https://www.cellsignal.cn/products/primary-antibodies/phospho-foxo1-thr24-foxo3a-thr32-antibody/9464?site-search-type=Products&N=4294956287&Ntt=phospho-foxo1+%28thr24%29%2Ffoxo3a&fromPage=plp, validated against starved cells
AMH (Rabbit polyclonal to AMH-C-terminal, abcam, #ab229212, 1:1000), https://www.abcam.com/amh-antibody-c-terminal-ab229212.html, validated against knockout.
AMH (Mouse monoclonal antibody, Santa Cruz Biotechnology, #sc-6886, 1:400), https://www.scbt.com/p/mis-antibody-c-20?requestFrom=search, validated against non-transfected cells.
FSHR (Rabbit polyclonal antibody, Bioworld, # BS6610, 1:1000),
https://www.bioworlde.com/FSHR-polyclonal-antibody(BS6610).html,validated against various cell lines.
Bcl-2 (Mouse monoclonal antibody, Santa Cruz Biotechnology, #sc-7382, 1:800),
https://www.scbt.com/p/bcl-2-antibody-c-2?requestFrom=search.
HRP conjugated rabbit IgG (Cell Signaling Technology, #7074, 1:4000), https://www.cellsignal.com/products/secondaryantibodies/anti-rabbit-igg-hrp-linked-antibody/7074
mouse IgG (Abcam, #ab131368, 1:1000; Cell Signaling Technology, #7076, 1:4000), https://www.cellsignal.com/products/secondary-antibodies/anti-mouse-igg-hrp-linked-antibody/7076
Alexa Fluor 546 conjugated goat anti-mouse secondary antibody (Thermo Fisher Scientific, # A-11030, 2 μg/ml).
https://www.thermofisher.com/cn/zh/antibody/product/Goat-anti-Mouse-IgG-H-L-Highly-Cross-Adsorbed-Secondary-Antibody-Polyclonal/A-11030
Alexa Fluor 488 conjugated goat anti-rabbit secondary antibody (Thermo Fisher Scientific, # A-11008, 2 μg/ml),
https://www.thermofisher.com/cn/zh/antibody/product/Goat-anti-Rabbit-IgG-H-L-Cross-Adsorbed-Secondary-Antibody-Polyclonal/

A-11008
BDNF (Rabbit monoclonal antibody, abcam, #ab108319, 1:100),
https://www.abcam.com/bdnf-antibody-epr1292-ab108319.html

Eukaryotic cell lines

Policy information about cell lines

Cell line source(s)	KGN cell line, a gift from Dr. Yi-Ming Mu, was originally derived from a patient with invasive ovarian granulosa cell carcinoma. Dr. Mu is one of the co-authors of the original paper. Reference: Nishi, Y. et al. Establishment and characterization of a steroidogenic human granulosa-like tumor cell line, KGN, that expresses functional follicle-stimulating hormone receptor. Endocrinology 142, 437-445 (2001).
Authentication	KGN cells were only authenticated by the morphology, and no further authentication method was used.
Mycoplasma contamination	All used cell lines tested negative for mycoplasma contamination
Commonly misidentified lines (See ICLAC register)	No commonly misidentified cell lines were used

Animals and other organisms

Policy information about studies involving animals; ARRIVE guidelines recommended for reporting animal research

Laboratory animals	Female (15-day-old, 6-8 months and 6-8 weeks old) and male (6-8 weeks old) C57BL/6 mice were purchased from Beijing Vital River Laboratory Animal Technology Co., Ltd. and housed in the specific pathogen free (SPF) condition with temperature control (22±1°C) and humidity control (60±10%) on a 12 hours light/12 hours dark cycle with ad libitum access to water and a regular rodent chow.
Wild animals	This study did not involve wild animals.
Field-collected samples	No field collection of samples was conducted in this study.
Ethics oversight	All the animal protocols were approved by Tsinghua University Animal Care and Use Committee.

Note that full information on the approval of the study protocol must also be provided in the manuscript.

Human research participants

Policy information about studies involving human research participants

Population characteristics	Ovarian sections were from seven female patients who underwent ovariectomy or hysterectomy. All the donors ranged in age from 20 to 64 years, with no history of any autoimmune or genetic disease. They were divided into two groups:the young (ages 29-35) and aged (61-64) groups. These donors did not undergo hormonal therapy at least 6 months before surgery, and were not exposed to any cytotoxic agents or radiotherapy. No ovary samples exhibited any histopathological abnormality, as was confirmed by gynecological pathologists.<br>To examine TrkB sigaling, human ovarian tissues were from female donors (ages 40-50) who underwent surgery for endometrial stromal sarcoma.
Recruitment	With written informed consent, participants were recruited by the clinician. There is no any self-selection bias in the recruitment.
Ethics oversight	This study conformed to the Declaration of Helsinki and the protocol was approved by the Ethics Committee of Beijing Obstetrics and Gynecology Hospital. Participants were compensated for their participation.

Note that full information on the approval of the study protocol must also be provided in the manuscript.
